# Red Light-Emitting Diode Based on Blue InGaN Chip with CdTe_*x*_S_(1 − *x*)_ Quantum Dots

**DOI:** 10.1186/s11671-016-1814-x

**Published:** 2017-01-19

**Authors:** Rongfang Wang, Xingming Wei, Liqin Qin, Zhihui Luo, Chunjie Liang, Guohang Tan

**Affiliations:** 1 Guangxi Key Laboratory of Agricultural Products Processing (Cultivating Base), Colleges and Universities Key Laboratory for Efficient Use of Agricultural Resources in the Southeast of Guangxi, College of Chemistry and Food Science, Yulin Normal University, Yulin, Guangxi 537000 People’s Republic of China; 2 Guangxi Experiment Center of Information Science, Guilin, People’s Republic of China

**Keywords:** Luminescence, Optical materials and properties, Semiconductors

## Abstract

Thioglycolic acid-capped CdTe_*x*_S_(1 − *x*)_ quantum dots (QDs) were synthesized through a one-step approach in an aqueous medium. The CdTe_*x*_S_(1 − *x*)_ QDs played the role of a color conversion center. The structural and luminescent properties of the obtained CdTe_*x*_S_(1 − *x*)_ QDs were investigated. The fabricated red light-emitting hybrid device with the CdTe_*x*_S_(1 − *x*)_ QDs as the phosphor and a blue InGaN chip as the excitation source showed a good luminance. The Commission Internationale de L’Eclairage coordinates of the light-emitting diode (LED) at (0.66, 0.29) demonstrated a red LED. Results showed that CdTe_*x*_S_(1 − *x*)_ QDs can be excited by blue or near-UV regions. This feature presents CdTe_*x*_S_(1 − *x*)_ QDs with an advantage over wavelength converters for LEDs.

## Background

One of the main challenges in communication and illumination industries is the development of full color displays and solid-state light-emitting devices. White light-emitting diodes (LEDs), which are considered as the next-generation solid-state illuminants, have recently gained considerable attention because of their high efficiency, long service life, and environmental protection [1–3]. At present, white LEDs (WLEDs) are fabricated by combining blue-emitting InGaN chips with yellow-emitting Ce^3+^-doped Y_3_Al_5_O_12_ phosphors. However, Ce^3+^-doped Y_3_Al_5_O_12_ phosphor-based WLEDs have certain disadvantages, such as low luminous efficiency and a poor color rendering index owing to their red spectral deficiency [4–7]. A variety of red phosphors have been studied to increase red emissions [8–13]. Among the various new red phosphors, II–VI or III–V semiconductor nanoparticles have been widely investigated for wavelength converters [14, 15]. Compared with binary quantum dots (QDs) (CdSe, ZnSe, CdTe, etc.), ternary alloy QDs have received a great deal of attention because they can be used in device fields because of their photoluminescence (PL) properties that can be tuned by controlling particle size and the composition of the alloy QDs [16, 17]. Cadmium sulfur (CdS) is one of the most important group II–VI nanoparticle (NC) semiconductors and displays a wide direct bandgap (2.42 eV). Compared with CdSe, CdTe QDs have greater exciton Bohr radius (7.3 nm) and stronger quantum size effect [18]. The band gap and lattice parameters of CdTe_*x*_S_(1 − *x*)_ ternary alloy QDs can be varied by adjusting the concentration of S and Te in the CdTe_*x*_S_(1 − *x*)_ compound. In addition, semiconductor nanoparticles can be excited by any optical source with an energy larger than their exciton energy [19].

In the present study, a facile method was developed to synthesize water-soluble red-emitting CdTe_*x*_S_(1 − *x*)_ alloyed QDs by using thioglycolic acid (TGA) as a stabilizer. Compared with the traditional two step aqueous synthesis, the approach proposed in the current study is simpler and more environment-friendly. The effects of reaction time and Te:S mole ratio on the maximum emission wavelength, full width at half maximum (FWHM), and PL quantum yield (QY) were also investigated. Furthermore, a red LED was fabricated by combining a 460-nm emitting InGaN chip with CdTe_*x*_S_(1 − *x*)_ NCs. The performance of the fabricated red LED was then evaluated.

## Methods

The red-emitting CdTe_*x*_S_(1 − *x*)_ QDs were synthesized through a one-step approach in an aqueous medium using TeO_2_, Na_2_S, NaBH_4_, and CdCl_2_·2.5H_2_O as precursors. Exactly 0.3 mL TGA and 100 mL CdCl_2_·2.5H_2_O solution were added to 250 mL of three-necked flask solution and then mixed under stirring. The solution was then adjusted to pH 10.5 with the dropwise addition of 1 mol/L NaOH solution. TeO_2_, Na_2_S, and NaBH_4_ were then injected into the original solution under stirring. The resulting mixture solution was heated to 100 °C and refluxed for different periods to control the size of the CdTe_*x*_S_(1 − *x*)_ QDs. These CdTe_*x*_S_(1 − *x*)_ NCs were precipitated with the excess absolute ethyl alcohol added to the CdTe_*x*_S_(1 − *x*)_ QD aqueous solution, centrifuged, and then dried at room temperature.

PL and UV-Vis absorption spectra were measured using a FluoroMax-4 fluorescence spectrometer and Cary 5000 spectrometer, respectively. The PL QY was determined using Rhodamine 6G as reference. High-resolution transmission electron microscopy (HRTEM) images were obtained with Tecnai G2 F20. X-ray diffraction (XRD) analysis was performed using Rigaku/Dmax-2500 (Cu Kα = 1.5406 Å). LED parameters were measured in an integrating sphere, which was connected to a CCD detector (HAAS-1200) under 20 mA forward bias.

## Results and Discussion

**Fig. 1:**
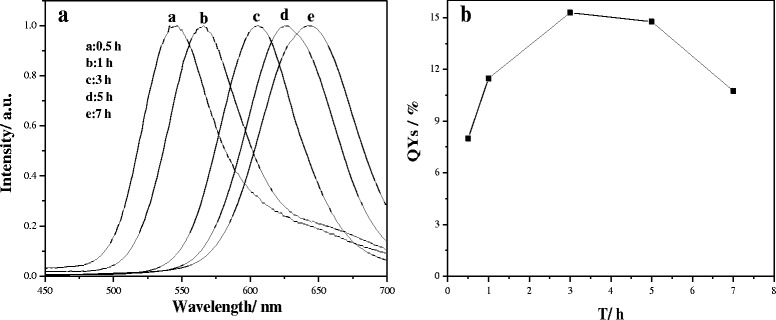
PL spectra (**a**) and the corresponding PL QYs (**b**) of CdTe_*x*_S_(1 − *x*)_ QDs at different reflux times

The effect of reflux time on the optical properties of the CdTe_*x*_S_(1 − *x*)_ QDs was investigated. Figure 1 shows the PL and corresponding QY of the CdTe_*x*_S_(1 − *x*)_ QDs for different reflux times (varying from 0.5 h to 7 h). The Te:S molar ratio was 0.3:1.7, and the temperature of the system was maintained at 100 °C. With an increase in reaction time from 0.5 to 7 h, the maximum emission peak exhibited an evident red shift from 544 nm to a long wavelength of 644 nm because of the quantum confinement effect. The size of CdTe_*x*_S_(1 − *x*)_ QDs grown at different reaction times was measured by 3D LS Spectrometer. The size of particles is 3.87, 3.98, 4.18, 4.32, and 4.43 nm. This is further evidence that the particle size of CdTe_*x*_S_(1 − *x*)_ QDs increases as the prolonged reaction times. The FWHM of the PL spectra was between 64 and 81 nm. As shown in Fig. 1b, the QYs of the CdTe_*x*_S_(1 − *x*)_ QDs initially increased and then decreased with increasing reflux time. The highest QYs reached 15.29% when the reflux time was 3 h.

**Fig. 2:**
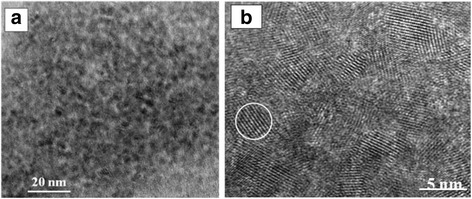
TEM (**a**) and HRTEM (**b**) images of CdTe_*x*_S_(1 − *x*)_ QDs

**Fig. 3:**
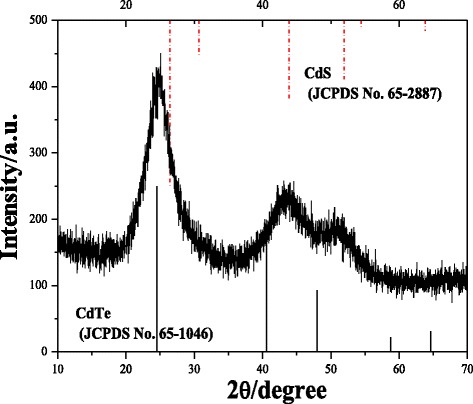
XRD pattern of CdTe_*x*_S_(1 − *x*)_ QDs

The transmission electron microscopy and HRTEM images in Fig. 2 clearly show nearly monodispersed particles, which are approximately spherical with an average diameter of approximately 4 nm. The lattice fringes of an individual particle indicating the highly crystalline structure of the CdTe_*x*_S_(1 − *x*)_ NCs are shown in the HRTEM image. The XRD pattern of the corresponding CdTe_*x*_S_(1 − *x*)_ NCs is shown in Fig. 3. The references of the bulk CdTe cubic structure (JCPDS No. 65-1046) and the bulk CdS cubic structure (JCPDS No. 65-2887) are also provided in this image for comparison. The XRD diffraction peak of the CdTe_*x*_S_(1 − *x*)_ NCs was located between those of the CdS and CdTe NCs, thereby confirming the formation of the CdTe_*x*_S_(1 − *x*)_ alloy QD NCs [20].

**Fig. 4:**
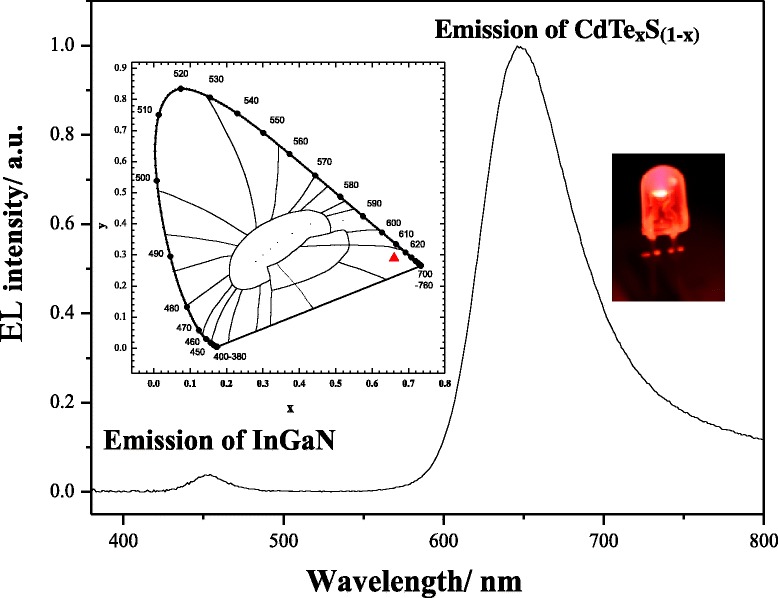
EL spectra of CdTe_*x*_S_(1 − *x*)_ QDs. *Insets* CIE color chromaticity diagram and photographs of the respective LEDs under a working current of 20 mA

An LED was fabricated by coating the CdTe_*x*_S_(1 − *x*)_ NCs onto a 460-nm emitting InGaN chip. The electroluminescence (EL) spectra of the red LED with CdTe_*x*_S_(1 − *x*)_ NCs under 20 mA forward bias are shown in Fig. 4. In Fig. 4, two emission bands can be observed at 460 and 647 nm. The peak at 647 nm was due to the emissions of the CdTe_*x*_S_(1 − *x*)_ NCs. The blue emission at 460 nm was attributed to the emission of the InGaN chip because the CdTe_*x*_S_(1 − *x*)_ NCs were unable to absorb the whole 460-nm emission from the underlying blue LED chip. The residual 460-nm LED chip emission may be used as the excitation source of other phosphors (green phosphors). Thus, a three-band WLED can be fabricated by combining a 460-nm emitting InGaN chip with CdTe_*x*_S_(1 − *x*)_ NCs (as a red phosphor) and the appropriate green phosphors. The insets in Fig. 4 show a photograph and the corresponding Commission Internationale de L’Eclairage (CIE) coordinates of the corresponding red LED under 20 mA forward bias. As shown in the figure, the CIE color coordinates of the device are (0.66, 0.29). The red light generated in the work showed a low color temperature of 1001 K. This result indicates that CdTe_*x*_S_(1 − *x*)_ QDs are good candidates for LED applications.

## Conclusions

CdTe_*x*_S_(1 − *x*)_ QDs (QYs = 15.29%) with tunable emission wavelengths were successfully synthesized in an aqueous solution by delicately controlling the reaction time. With an increase in reaction time, the PL emission peak exhibited an evident red shift. The HRTEM results showed that the size of the CdTe_*x*_S_(1 − *x*)_ QDs was approximately 4 nm. The QDs were successfully used for phosphors for LEDs. CdTe_*x*_S_(1 − *x*)_ NCs and a 460-nm emitting InGaN chip were fabricated to test the EL properties. The CIE coordinates of the red LED were located at (0.66, 0.29).

## Abbreviations

CIE: Commission Internationale de L’Eclairage
EL: Electroluminescence
FWHM: Full width at half maximum
HRTEM: High-resolution transmission electron microscopy
LEDs: Light-emitting diodes
NCs: Nanoparticles
PL: Photoluminescence
QDs: Quantum dots
QY: Quantum yield
TGA: Thioglycolic acid
WLEDs: White light-emitting diode
XRD: X-ray diffraction
